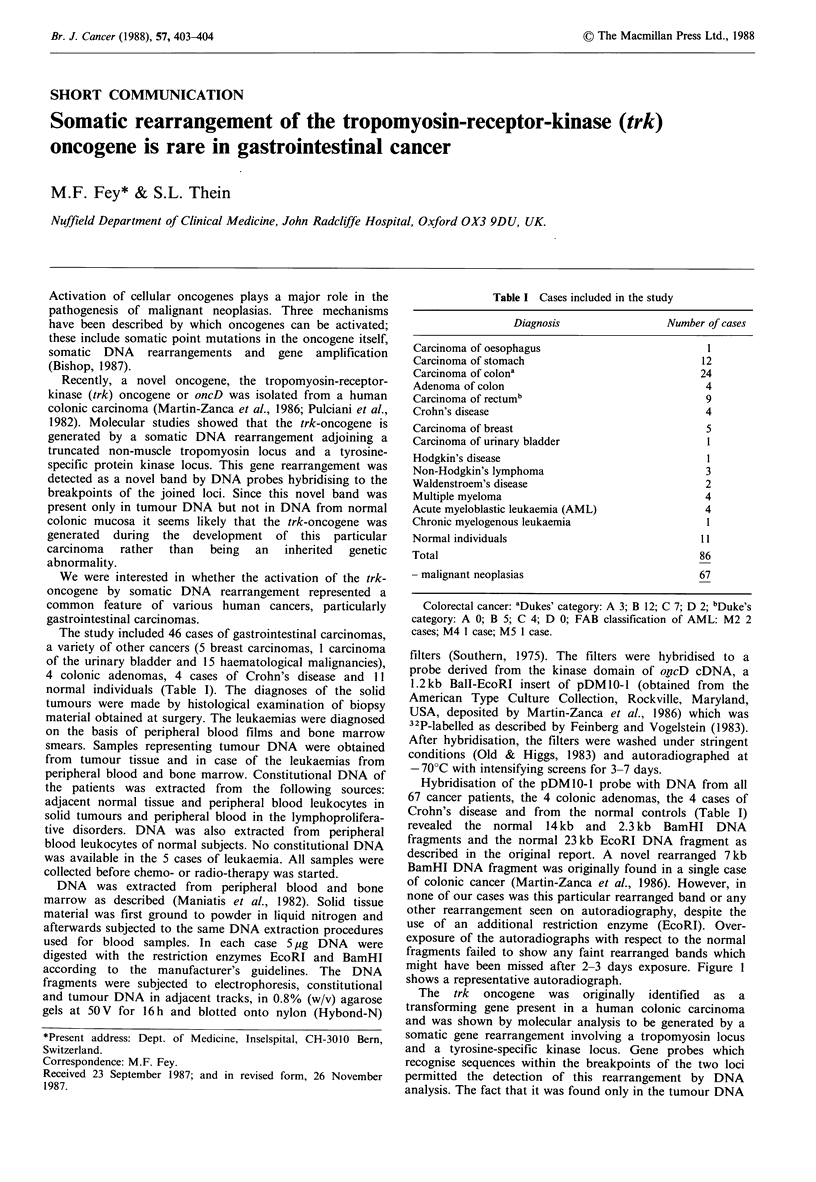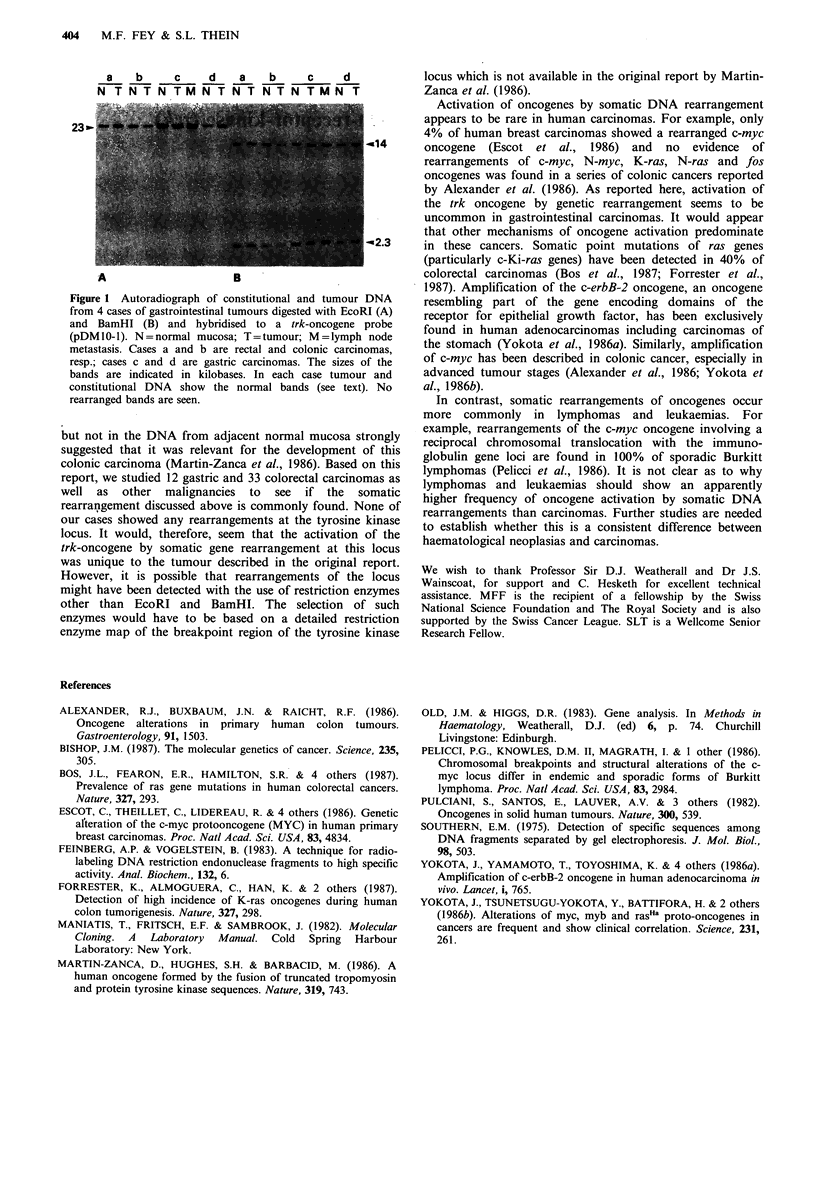# Somatic rearrangement of the tropomyosin-receptor-kinase (trk) oncogene is rare in gastrointestinal cancer.

**DOI:** 10.1038/bjc.1988.90

**Published:** 1988-04

**Authors:** M. F. Fey, S. L. Thein

**Affiliations:** Nuffield Department of Clinical Medicine, John Radcliffe Hospital, Oxford, UK.

## Abstract

**Images:**


*Br. J. Cancer* (1988), **57**, 403–404 © The Macmillan Press Ltd., 1988

SHORT COMMUNICATION

### Somatic rearrangement of the tropomyosin-receptor-kinase (*trk*) oncogene is rare in gastrointestinal cancer

M.F. Fey* & S.L. Thein

*Nuffield Department of Clinical Medicine, John Radcliffe Hospital, Oxford OX3 9DU, UK.*

Activation of cellular oncogenes plays a major role in the pathogenesis of malignant neoplasias. Three mechanisms have been described by which oncogenes can be activated; these include somatic point mutations in the oncogene itself, somatic DNA rearrangements and gene amplification (Bishop, 1987).

Recently, a novel oncogene, the tropomyosin-receptor-kinase (*trk*) oncogene or *oncD* was isolated from a human colonic carcinoma (Martin-Zanca *et al.*, 1986; Pulciani *et al.*, 1982). Molecular studies showed that the *trk*-oncogene is generated by a somatic DNA rearrangement adjoining a truncated non-muscle tropomyosin locus and a tyrosine-specific protein kinase locus. This gene rearrangement was detected as a novel band by DNA probes hybridising to the breakpoints of the joined loci. Since this novel band was present only in tumour DNA but not in DNA from normal colonic mucosa it seems likely that the *trk*-oncogene was generated during the development of this particular carcinoma rather than being an inherited genetic abnormality.

We were interested in whether the activation of the *trk*-oncogene by somatic DNA rearrangement represented a common feature of various human cancers, particularly gastrointestinal carcinomas.

The study included 46 cases of gastrointestinal carcinomas, a variety of other cancers (5 breast carcinomas, 1 carcinoma of the urinary bladder and 15 haematological malignancies), 4 colonic adenomas, 4 cases of Crohn's disease and 11 normal individuals (Table I). The diagnoses of the solid tumours were made by histological examination of biopsy material obtained at surgery. The leukaemias were diagnosed on the basis of peripheral blood films and bone marrow smears. Samples representing tumour DNA were obtained from tumour tissue and in case of the leukaemias from peripheral blood and bone marrow. Constitutional DNA of the patients was extracted from the following sources: adjacent normal tissue and peripheral blood leukocytes in solid tumours and peripheral blood in the lymphoproliferative disorders. DNA was also extracted from peripheral blood leukocytes of normal subjects. No constitutional DNA was available in the 5 cases of leukaemia. All samples were collected before chemo- or radio-therapy was started.

DNA was extracted from peripheral blood and bone marrow as described (Maniatis *et al.*, 1982). Solid tissue material was first ground to powder in liquid nitrogen and afterwards subjected to the same DNA extraction procedures used for blood samples. In each case 5 μg DNA were digested with the restriction enzymes EcoRI and BamHI according to the manufacturer's guidelines. The DNA fragments were subjected to electrophoresis, constitutional and tumour DNA in adjacent tracks, in 0.8% (w/v) agarose gels at 50 V for 16 h and blotted onto nylon (Hybond-N) filters (Southern, 1975). The filters were hybridised to a probe derived from the kinase domain of oncD cDNA, a 1.2 kb BalI-EcoRI insert of pDM10-1 (obtained from the American Type Culture Collection, Rockville, Maryland, USA, deposited by Martin-Zanca *et al.*, 1986) which was $^{32}P$-labelled as described by Feinberg and Vogelstein (1983). After hybridisation, the filters were washed under stringent conditions (Old & Higgs, 1983) and autoradiographed at −70°C with intensifying screens for 3–7 days.

**Table I** Cases included in the study

| *Diagnosis* | *Number of cases* |
|---|---|
| Carcinoma of oesophagus | 1 |
| Carcinoma of stomach | 12 |
| Carcinoma of colon[a] | 24 |
| Adenoma of colon | 4 |
| Carcinoma of rectum[b] | 9 |
| Crohn's disease | 4 |
| Carcinoma of breast | 5 |
| Carcinoma of urinary bladder | 1 |
| Hodgkin's disease | 1 |
| Non-Hodgkin's lymphoma | 3 |
| Waldenstroem's disease | 2 |
| Multiple myeloma | 4 |
| Acute myeloblastic leukaemia (AML) | 4 |
| Chronic myelogenous leukaemia | 1 |
| Normal individuals | 11 |
| Total | 86 |
| – malignant neoplasias | 67 |

Colorectal cancer: [a]Dukes' category: A 3; B 12; C 7; D 2; [b]Duke's category: A 0; B 5; C 4; D 0; FAB classification of AML: M2 2 cases; M4 1 case; M5 1 case.

Hybridisation of the pDM10-1 probe with DNA from all 67 cancer patients, the 4 colonic adenomas, the 4 cases of Crohn's disease and from the normal controls (Table I) revealed the normal 14 kb and 2.3 kb BamHI DNA fragments and the normal 23 kb EcoRI DNA fragment as described in the original report. A novel rearranged 7 kb BamHI DNA fragment was originally found in a single case of colonic cancer (Martin-Zanca *et al.*, 1986). However, in none of our cases was this particular rearranged band or any other rearrangement seen on autoradiography, despite the use of an additional restriction enzyme (EcoRI). Over-exposure of the autoradiographs with respect to the normal fragments failed to show any faint rearranged bands which might have been missed after 2–3 days exposure. Figure 1 shows a representative autoradiograph.

The *trk* oncogene was originally identified as a transforming gene present in a human colonic carcinoma and was shown by molecular analysis to be generated by a somatic gene rearrangement involving a tropomyosin locus and a tyrosine-specific kinase locus. Gene probes which recognise sequences within the breakpoints of the two loci permitted the detection of this rearrangement by DNA analysis. The fact that it was found only in the tumour DNA

*Present address: Dept. of Medicine, Inselspital, CH-3010 Bern, Switzerland.
Correspondence: M.F. Fey.
Received 23 September 1987; and in revised form, 26 November 1987.

**Figure 1** Autoradiograph of constitutional and tumour DNA from 4 cases of gastrointestinal tumours digested with EcoRI (A) and BamHI (B) and hybridised to a *trk*-oncogene probe (pDM10-1). N = normal mucosa; T = tumour; M = lymph node metastasis. Cases a and b are rectal and colonic carcinomas, resp.; cases c and d are gastric carcinomas. The sizes of the bands are indicated in kilobases. In each case tumour and constitutional DNA show the normal bands (see text). No rearranged bands are seen.

but not in the DNA from adjacent normal mucosa strongly suggested that it was relevant for the development of this colonic carcinoma (Martin-Zanca *et al.*, 1986). Based on this report, we studied 12 gastric and 33 colorectal carcinomas as well as other malignancies to see if the somatic rearrangement discussed above is commonly found. None of our cases showed any rearrangements at the tyrosine kinase locus. It would, therefore, seem that the activation of the *trk*-oncogene by somatic gene rearrangement at this locus was unique to the tumour described in the original report. However, it is possible that rearrangements of the locus might have been detected with the use of restriction enzymes other than EcoRI and BamHI. The selection of such enzymes would have to be based on a detailed restriction enzyme map of the breakpoint region of the tyrosine kinase locus which is not available in the original report by Martin-Zanca *et al.* (1986).

Activation of oncogenes by somatic DNA rearrangement appears to be rare in human carcinomas. For example, only 4% of human breast carcinomas showed a rearranged c-*myc* oncogene (Escot *et al.*, 1986) and no evidence of rearrangements of c-*myc*, N-*myc*, K-*ras*, N-*ras* and *fos* oncogenes was found in a series of colonic cancers reported by Alexander *et al.* (1986). As reported here, activation of the *trk* oncogene by genetic rearrangement seems to be uncommon in gastrointestinal carcinomas. It would appear that other mechanisms of oncogene activation predominate in these cancers. Somatic point mutations of *ras* genes (particularly c-Ki-*ras* genes) have been detected in 40% of colorectal carcinomas (Bos *et al.*, 1987; Forrester *et al.*, 1987). Amplification of the c-*erbB-2* oncogene, an oncogene resembling part of the gene encoding domains of the receptor for epithelial growth factor, has been exclusively found in human adenocarcinomas including carcinomas of the stomach (Yokota *et al.*, 1986*a*). Similarly, amplification of c-*myc* has been described in colonic cancer, especially in advanced tumour stages (Alexander *et al.*, 1986; Yokota *et al.*, 1986*b*).

In contrast, somatic rearrangements of oncogenes occur more commonly in lymphomas and leukaemias. For example, rearrangements of the c-*myc* oncogene involving a reciprocal chromosomal translocation with the immunoglobulin gene loci are found in 100% of sporadic Burkitt lymphomas (Pelicci *et al.*, 1986). It is not clear as to why lymphomas and leukaemias should show an apparently higher frequency of oncogene activation by somatic DNA rearrangements than carcinomas. Further studies are needed to establish whether this is a consistent difference between haematological neoplasias and carcinomas.

We wish to thank Professor Sir D.J. Weatherall and Dr J.S. Wainscoat, for support and C. Hesketh for excellent technical assistance. MFF is the recipient of a fellowship by the Swiss National Science Foundation and The Royal Society and is also supported by the Swiss Cancer League. SLT is a Wellcome Senior Research Fellow.

### References

ALEXANDER, R.J., BUXBAUM, J.N. & RAICHT, R.F. (1986). Oncogene alterations in primary human colon tumours. *Gastroenterology*, **91**, 1503.

BISHOP, J.M. (1987). The molecular genetics of cancer. *Science*, **235**, 305.

BOS, J.L., FEARON, E.R., HAMILTON, S.R. & 4 others (1987). Prevalence of ras gene mutations in human colorectal cancers. *Nature*, **327**, 293.

ESCOT, C., THEILLET, C., LIDEREAU, R. & 4 others (1986). Genetic alteration of the c-myc protooncogene (MYC) in human primary breast carcinomas. *Proc. Natl Acad. Sci. USA*, **83**, 4834.

FEINBERG, A.P. & VOGELSTEIN, B. (1983). A technique for radiolabeling DNA restriction endonuclease fragments to high specific activity. *Anal. Biochem.*, **132**, 6.

FORRESTER, K., ALMOGUERA, C., HAN, K. & 2 others (1987). Detection of high incidence of K-ras oncogenes during human colon tumorigenesis. *Nature*, **327**, 298.

MANIATIS, T., FRITSCH, E.F. & SAMBROOK, J. (1982). *Molecular Cloning. A Laboratory Manual*. Cold Spring Harbour Laboratory: New York.

MARTIN-ZANCA, D., HUGHES, S.H. & BARBACID, M. (1986). A human oncogene formed by the fusion of truncated tropomyosin and protein tyrosine kinase sequences. *Nature*, **319**, 743.

OLD, J.M. & HIGGS, D.R. (1983). Gene analysis. In *Methods in Haematology*, Weatherall, D.J. (ed) **6**, p. 74. Churchill Livingstone: Edinburgh.

PELICCI, P.G., KNOWLES, D.M. II, MAGRATH, I. & 1 other (1986). Chromosomal breakpoints and structural alterations of the c-myc locus differ in endemic and sporadic forms of Burkitt lymphoma. *Proc. Natl Acad. Sci. USA*, **83**, 2984.

PULCIANI, S., SANTOS, E., LAUVER, A.V. & 3 others (1982). Oncogenes in solid human tumours. *Nature*, **300**, 539.

SOUTHERN, E.M. (1975). Detection of specific sequences among DNA fragments separated by gel electrophoresis. *J. Mol. Biol.*, **98**, 503.

YOKOTA, J., YAMAMOTO, T., TOYOSHIMA, K. & 4 others (1986*a*). Amplification of c-erbB-2 oncogene in human adenocarcinoma *in vivo*. *Lancet*, **i**, 765.

YOKOTA, J., TSUNETSUGU-YOKOTA, Y., BATTIFORA, H. & 2 others (1986*b*). Alterations of myc, myb and $ras^{Ha}$ proto-oncogenes in cancers are frequent and show clinical correlation. *Science*, **231**, 261.